# Cognitive and Psychosocial Burden of Childhood Cancer Survivors in Greece: A Case–Control Study

**DOI:** 10.3390/medsci14020171

**Published:** 2026-03-30

**Authors:** Kalliopi Mavrea, Katerina Katsibardi, Kleoniki Roka, Roser Pons, Vasiliki Efthymiou, Alexandros-Stamatios Antoniou, Antonios I. Christou, Christina Kanaka-Gantenbein, George P. Chrousos, Antonis Kattamis, Flora Bacopoulou

**Affiliations:** 1 Clinic for Assessment of Learning Difficulties, Center for Adolescent Medicine and UNESCO Chair in Adolescent Health Care, First Department of Pediatrics, Medical School, National and Kapodistrian University of Athens, Aghia Sophia Children’s Hospital, 1 Thivon Street, 11527 Athens, Greece; 2 University Oncology Hematology Unit, First Department of Pediatrics, Medical School, National and Kapodistrian University of Athens, Aghia Sophia Children’s Hospital, 1 Thivon Street, 11527 Athens, Greece; 3 Pediatric Neurology Unit, First Department of Pediatrics, Medical School, National and Kapodistrian University of Athens, Aghia Sophia Children’s Hospital, 1 Thivon Street, 11527 Athens, Greece; 4 University Research Institute for the Study of Genetic and Malignant Disorders in Childhood, Medical School, National and Kapodistrian University of Athens, 8 Levadeias Street, 11527 Athens, Greece; 5 Faculty of Primary Education, National and Kapodistrian University of Athens, 20 Ippokratous Street, 10680 Athens, Greece; 6 Department of Special Education, University of Thessaly, 38221 Volos, Greece

**Keywords:** cancer survivors, Greece, cancer, cognitive, education, learning difficulties, IQ, psychological, quality of life, intelligence

## Abstract

Background/Objectives: To study the hypothesis that cognitive functions and learning skills are impaired in child/adolescent childhood cancer survivors (CCS). Secondary outcomes included psychosocial parameters and quality of life. Methods: This case–control study was conducted over four years (2017–2021) at the largest pediatric Aghia Sophia Children’s Hospital, in Greece. Eligible participants were children and adolescents in Greece. For CCS, ≥1 year should have elapsed from completion of cancer treatment. Assessments of neurocognitive function, learning and psychosocial skills and health-related quality of life (HRQoL) were performed with validated instruments (WISC-III, LAMDA software, Achenbach CBCL/6-18 and YSR, KIDSCREEN-52, respectively). Results: In total, 219 participants (47.49% males, mean age ± SD 11.72 ± 2.32 years), 70 CCS and 149 controls (matched for age, sex, family income), were included. Cases were CCS of acute lymphoblastic leukemia (*n* = 25)/brain tumors (*n* = 19)/lymphoma (*n* = 17)/nephroblastoma (*n* = 5)/Ewing sarcoma (*n* = 3)/rhabdomyosarcoma (*n* = 1). CCS had worse scores in full-scale Intelligence Quotient (FSIQ) (*p* = 0.004), verbal IQ (VIQ) (*p* = 0.005) and all its subscales, performance IQ (PIQ) (*p* = 0.021), and almost all learning parameters than controls. Attention, working memory, writing/visual–motor coordination, processing accuracy/speed, language acquisition/expression, all psychosocial scales, and HRQoL domains of mood and emotions, were negatively affected in CCS. Female CCS demonstrated lower FSIQ (*p* = 0.019) and VIQ (*p* = 0.014) than control females, whereas male CCS retained their total IQ unaffected. Among CCS, those with non-central nervous system (CNS) tumors, higher parental educational level or higher family income had significantly higher IQ than those with CNS tumors, lower parental educational level or lower family income, respectively. Conclusions: CCS in Greece carry a significant burden of cognitive and psychological morbidity. Cognitive/educational and psychosocial support to CCS is imperative.

## 1. Introduction

Cancer treatments, including chemotherapy, radiotherapy and surgery, as well as other advanced modalities, have resulted in significant improvements in survival rates in childhood cancer in high-income countries. However, in some cases, high doses of chemotherapy and/or radiotherapy and aggressive surgery have been associated with severe morbidity. Added adversities of cancer treatment in childhood cancer survivors (CCS) include neurocognitive dysfunction. Chemotherapy and radiotherapy have been linked to CCS’ “brain fog”, i.e., different degrees of cognitive dysfunction (rather than global retardation) via cytotoxic effects and injury to the central nervous system (CNS) [1,2,3,4]. In some studies, treatment-related exposures were not associated with neurocognitive function but with lower parental educational attainment, unemployment, and low occupational status [2]. A small number of children may achieve cognitive or academic outcomes comparable to or higher than expected based on their clinical history, indicating that individualized tutoring and parental support may reduce some learning deficits [5].

Childhood and adolescence represent critical developmental stages during which rapid cognitive, emotional, and social maturation occurs. Disruptions during this period—such as those caused by severe illnesses, including cancer—may have long-lasting implications for an individual’s developmental trajectory. A growing body of international research has demonstrated that CCS are at increased risk of neurocognitive difficulties, including impairments in attention, processing speed, working memory, and executive functioning, which may persist long after completion of treatment and influence academic performance and everyday functioning [6].

Beyond cognitive outcomes, childhood cancer may also affect broader psychosocial and socioeconomic development. Studies from Europe and North America have shown that CCS may experience disruptions in schooling, delayed educational progression, and increased need for special educational support compared with their peers [7]. Despite significant improvements in survival rates over recent decades, the long-term developmental and psychosocial consequences of childhood cancer remain an important public health concern. Children and adolescents with cancer therefore constitute a distinct population with unique medical, psychological, and social needs whose long-term outcomes should be systematically assessed. When estimating the burden of cancer, however, these developmental aspects are often overlooked, even though cancer survivorship may have profound implications for childhood and adolescent education, relationships, and future career trajectories.

To our knowledge, there are no published studies examining the cognitive and learning outcomes of child/adolescent CCS in Greece.

Thus, the primary aim of this study was to assess potential differences in cognitive functions and learning skills between child/adolescent CCS and controls, with regard to the type of neoplasia, its treatment, time free of cancer, and CCS birth weight, mode of delivery, and body mass index (BMI). Secondary outcomes assessed included psychosocial parameters and quality of life.

## 2. Materials and Methods

### 2.1. Study Design Setting

This case–control study assessed the cognitive outcomes among CCS who had been treated for any type of cancer at the Pediatric Hematology Oncology Unit of the Fist Department of Pediatrics, Medical School, National and Kapodistrian University of Athens, at the tertiary Aghia Sophia Children’s Hospital, the largest pediatric hospital in Greece. The study was conducted over a period of four years (February 2017 to February 2021) and in accordance with the Declaration of Helsinki. Approval was acquired from the Aghia Sophia Children’s Hospital Research Ethics Committee (protocol number 2621/23-02-17). Reporting of the study conforms to the STROBE statement [8].

### 2.2. Participants

Participants included in this analysis were children and adolescents in Greece.

For survivors, a period of ≥1 year was required to have elapsed since completion of cancer treatment to ensure that participants had transitioned beyond the acute treatment phase and into the survivorship period (“short-term cancer survivors”), where longer-term outcomes and treatment-related effects can be more appropriately evaluated [9]. Exclusion criteria included global intellectual impairment, i.e., intelligence quotient (IQ) ≤ 70, CNS injury or attention deficit hyperactivity disorder predating cancer diagnosis, and current use of psychotropic medications. Individuals were also excluded if they suffered from a major mental health disorder or significant sensory/motor impairment that could impact assessment validity.

Parents or guardians of eligible CCS were contacted through telephone calls, informed about the study procedures, and invited to participate in the study. Those who were interested in participating were recruited and offered a further appointment for cognitive assessment at the Clinic for Assessment of Adolescent Learning Difficulties of the Center for Adolescent Medicine and UNESCO Chair on Adolescent Health Care of the Fist Department of Pediatrics, at the same health care provider, Aghia Sophia Children’s Hospital.

Healthy control participants who attended the Center for Adolescent Medicine were recruited during routine healthcare visits in order to meet the above exclusion criteria while ensuring similar access to healthcare services. All control participants were screened to confirm the absence of major chronic medical conditions that could influence cognitive or psychosocial functioning. Controls were recruited prospectively through a follow-up appointment for cognitive assessment. The ratio of cases to controls was 1:2 to add power to the study [10].

Parents or guardians were informed in detail about the procedures and objectives of the research and signed a written consent prior to their children’s enrolment in the study. Child/adolescent assent was also obtained before data collection.

### 2.3. Procedures

The evaluation included detailed demographic and medical history, complete physical examination, school/learning and neurocognitive performance, mental health and quality of life assessment.

Birth details (birth weight, mode of delivery), BMI or body surface area (BSA) at cancer diagnosis, type of cancer, treatment regimens, type of surgery, if performed, chemotherapy protocols, radiation dose exposure, health sequelae after therapy and time free of cancer were obtained from CCS’ medical records.

Participants’ parents/guardians were asked to fill in a questionnaire regarding their demographic characteristics such as age, residence, educational level, job, family income and health status. Participants’ educational profile (school performance, learning difficulties, learning interventions, special educational needs) was also documented.

### 2.4. Assessments

#### 2.4.1. Achenbach Child Behavior Checklist (CBCL/6-18) and Youth Self-Report (YSR)

Participants’ psychosocial assessment was performed with the Greek version of the Achenbach Child Behavior Checklist (CBCL/6-18), completed by parents, and the Youth Self-Report (YSR), completed by children or adolescents [11,12]. The 2001 revision of the CBCL (CBCL/6-18) completed by parents and the YSR (completed by children or adolescents) provided scores for internalizing and externalizing behaviors, made up of responses to eight syndrome scales (anxious/depressed, withdrawn/depressed, somatic complaints, social/thought/attention problems, and rule-breaking/aggressive behavior), for the past six months. Six DSM-oriented scales (affective/anxiety/somatic/oppositional defiant/conduct problems and attention deficit/hyperactivity disorder) were also assessed. Optional competence scales recorded information concerning a child’s activities, social relationships, school performance, and total competence. Scores could be interpreted as falling in the normal, borderline, or clinical behavior range, with higher scores indicating greater problems.

#### 2.4.2. Health-Related Quality of Life

The Health-Related Quality of Life (HRQoL) Questionnaire for Children and Adolescents aged 8–18 years (KIDSCREEN-52), already validated in 13 European countries, was used [13,14]. It assesses the degree of social skills and quality of life in 10 HRQoL dimensions: Physical (5 items), Psychological Well-being (6 items), Moods and Emotions (7 items), Self-Perception (5 items), Autonomy (5 items), Parent Relations and Home Life (6 items), Social Support and Peers (6 items), School Environment (6 items), Social Acceptance (Bullying) (3 items), and Financial Resources (3 items). Cronbach’s alpha internal consistency index of the Greek version ranges from 0.73 to 0.90.

#### 2.4.3. Neurocognitive Assessment

A multidisciplinary team, comprising pediatric hematologists oncologists, a pediatric neurologist, an adolescent medicine specialist, a special education teacher, a speech therapist, a social worker and trained psychologists, clinically evaluated all study participants on pre-arranged dates, along with their brain magnetic resonance imaging (MRI) reports, teachers’ reports, and validated instruments. Validated tools for participant cognitive and learning assessment were the following:

#### 2.4.4. Wechsler Intelligence Scale for Children—Third Edition (WISC-III)

Cognitive function was measured with the Hellenic (Greek) version of the Wechsler Intelligence Scale for Children—Third Edition (WISC-III) [15,16], which measures full-scale IQ (FSIQ), comprising verbal IQ (VIQ) and performance IQ (PIQ). The FSIQ score is calculated by the scores of 13 subscales forming the (a) VIQ score [Information (IN), Similarities (SM), Arithmetic (AR), Vocabulary (VC), Comprehension (CM), Digit Span (DS)] and (b) PIQ score [Picture Completion (PC), Coding (CD), Picture Arrangement (PA), Block Design (BD), Object Assembly (OA), Symbol Search (SS)].

#### 2.4.5. Computer-Based Software for Detection of Learning Skills and Weaknesses (LAMDA)

Screening for learning difficulties was performed with the computer-based software for detection of learning skills and weaknesses LAMDA (Ministry of National Education and Religious Affairs, Greece, 2007), validated in the Greek population [17]. LAMDA examines skills that are indicators of learning potential rather than measuring school performance, as cognitive performance can be considerably impacted by other parameters, i.e., processing speed. It assesses accuracy and speed regarding word/picture recognition, spelling, oral/reading comprehension, morphosyntactic awareness, vocabulary, short-term memory, non-verbal reasoning and rhythm sensitivity. Results are categorized into four zones, i.e., red, yellow, light green and dark green zones, indicating student performance within 0–10%, 10–25%, 25–50% and 50–100% in relation to his/her peers, respectively.

### 2.5. Statistical Methods

Continuous variables are presented as mean (M) ± standard deviation (SD), non-parametric variables as median and interquartile range, and qualitative variables as frequencies and percentages. Comparisons of continuous data were carried out with the Student *t*-test after checking homogeneity of variance or with the Mann–Whitney U test for non-parametric variables. Discrete variables were compared with Fisher’s exact test. Analysis of variance (ANOVA) or the Kruskal–Wallis test were used to examine differences between more than two comparing groups for parametric and non-parametric variables, respectively. Post hoc tests were carried out using Bonferroni correction. Statistical significance was set at *p* < 0.05. All analyses were carried out using SPSS software 25 version for Windows (IBM Statistical Package for Social Sciences for Windows, Version 25.0; IBM Corp, Armonk, NY, USA) and R Statistics software version 4.0.3 (R Core Team, 2020) using the PWR package. In addition to statistical significance, effect sizes were calculated and reported to provide a better estimate of the magnitude of group differences. Only participants who completed the full assessment battery were included in the final analyses. Participants with incomplete assessment data were excluded from the analytic sample. No imputation procedures were applied.

## 3. Results

The study sample consisted of two groups; CCS and healthy controls. Parents or guardians completed questionnaires, providing proxy information about participating children and adolescents. Two hundred and ninety-two records of CCS were examined for eligibility, from which 221 CCS were deemed eligible and were contacted to participate in the study. Regarding controls, 723 preadolescents and adolescents were screened for eligibility, of whom 397 were confirmed eligible for study entry. Finally, a total of 219 children and adolescents (70 CCS and 149 controls; 47.49% males; mean age ± SD 11.72 ± 2.32 years) completed all the assessments, and their data were included in the study analyses. A substantial number of eligible CCS and controls did not complete all assessments, as this required families to spend a significant amount of time at the hospital to do so; hence, they were excluded from the analysis. Participants were survivors of acute lymphoblastic leukemia (ALL, *n* = 25), brain tumors (BT, *n* = 19), lymphoma (*n* = 17), or other types of cancer, i.e., nephroblastoma (*n* = 5), Ewing sarcoma (*n* = 3), and rhabdomyosarcoma (*n* = 1). Controls were matched for age, sex and family income.

### 3.1. Neurocognitive Function and Learning Skills

Participants’ cognitive and learning scores are presented in Table 1. Relative to controls, CCS demonstrated significantly lower scores of FSIQ, VIQ and PIQ. These differences were basically attributed to all verbal subscales of WISC-III, whereas disparities in performance subscales were significant only for CD and SS. Similarly, CCS demonstrated significantly lower scores in almost all learning parameters of LAMDA (except for word/picture recognition, speed of vocabulary, and rhythm sensitivity) than controls.

CCS’ cognitive and learning parameters were also studied according to the type and area of cancer. Survivors of BT had significantly lower mean FSIQ and PIQ than ALL survivors or survivors of other types of cancer. Survivors of CNS tumors scored significantly lower than survivors of non-CNS tumors, in FSIQ, VIQ, PIQ and in several subscales (IN, CM, DS, CD, PA, SS), as well as in the accuracy of rhythm sensitivity (Table 2).

The cognitive burden was significant in female CCS who demonstrated lower scores in FSIQ (*p* = 0.019) and VIQ (*p* = 0.014) and its subscales (SM, *p* = 0.026; CM, *p* = 0.035; DS, *p* = 0.025) than control females; however, their PIQ was not significantly affected. Male CCS compared to control males were significantly affected in only one subscale of VIQ (IN, *p* = 0.040) and two subscales of PIQ (PA, *p* = 0.046; SS, *p* < 0.001), but their total IQ scores were unaffected. Among CCS, females scored significantly lower than males in the AR subscale of VIQ (*p* = 0.036). Likewise, male CCS, compared to controls, scored significantly lower in some learning indices, namely, in speed of spelling (*p* = 0.044), speed of oral/reading comprehension (*p* = 0.003) and speed of morphosyntactic awareness (*p* = 0.017), as well as in accuracy of non-verbal reasoning (*p* = 0.023). Female CCS, compared to controls, scored significantly lower in accuracy of oral/reading comprehension (*p* = 0.039) and accuracy of morphosyntactic awareness (*p* = 0.032).

### 3.2. Radiotherapy

Practical IQ in general and its subscale PA were significantly lower (*p* = 0.042; *p* = 0.020, respectively) for radiotherapy-treated than non-radiotherapy-treated CCS. Likewise, significantly (*p* = 0.029 and *p* = 0.044, respectively) lower scores for speed of oral/reading comprehension and non-verbal reasoning were observed in radiotherapy-treated vs. non-radiotherapy-treated CCS. The radiation load correlated negatively with the PIQ subscales, i.e., the number of radiation days with SS (r = −0.551, *p* = 0.027), whereas radiation dose correlated with CD (r = −0.556, *p* = 0.020). Survivors who received cranial/CNS radiation vs. other non-CNS body areas scored lower in the PIQ subscale of CD (M ± SD, 6.64 ± 3.79 vs. 10.71 ± 1.25, *p* = 0.002).

### 3.3. Surgery

Non-significant differences were found for all scales and subscales of WISC-III and LAMDA between CCS who underwent surgery (as the only treatment) and CCS who received non-surgical treatment.

### 3.4. Birth Weight, Mode of Delivery, BMI and Time Free of Cancer

Survivors’ birth weight correlated positively with PIQ (r = 0.401, *p* = 0.019) and its subscale of PA (r = 0.482, *p* = 0.004). Time free of cancer correlated positively with the VIQ subscale of IN (r = 0.359, *p* = 0.044). No correlations of the WISC-III and LAMDA scales/subscales were found with respect to the mode of delivery (vaginal or cesarian section), body mass index (BMI) or body surface area (BSA) at diagnosis, and BMI at the time of cognitive assessment.

### 3.5. Psychosocial Function

Parents of CCS, compared to control parents, scored significantly higher in their offspring internalizing, externalizing and overall problems, i.e., anxiety, depression, somatic problems, social problems, thinking problems, attention problems, breaking rules, and aggressive behavior, oppositional/defiant/conduct and attention deficit/hyperactivity problems, post-traumatic stress, and obsessive–compulsive problems.

Children’s results (YSR) coincided with those of their parents, while CCS demonstrated significantly worse scores in all the aforementioned scales than controls (Table 3).

### 3.6. Parental Education and Family Financial Status

Parental educational level and economic status (based on the yearly income cut-off of 20,000 €) were studied according to the cognitive and learning performance of CCS (Table 4). Survivors who had at least one parent of a higher educational level had higher scores in FSIQ, in VIQ and its subscales IN, SM, VC, and DS, and in the PIQ subscales of PA, BD, and SS, as well as in accuracy of word/picture recognition, oral/reading comprehension and morphosyntactic awareness, than CCS with parents of a secondary (high school graduates) or lower educational level. Furthermore, CCS of higher family income (yearly income > 20,000 €) scored significantly higher in the VIQ subscales of IN and DS and in the PIQ subscale of SS than CCS of families with lower income (≤20,000 €).

### 3.7. Health-Related Quality of Life

CCS, compared to controls, reported significantly lower mood and emotions but greater autonomy (Table 5).

## 4. Discussion

To our knowledge, this is the first study to examine the cognitive and learning outcomes of CCS in Greece. The study raises concerns not only about the neurocognitive function and learning skills of CCS, but also about their psychosocial function. CCS, compared to controls, had significantly worse scores in FSIQ and PIQ, in all verbal IQ subscales, in almost all learning parameters, in all psychosocial scales and in the HRQoL domains of mood and emotions. Neurobehavioral morbidity in our study affected several aspects of cognitive function, which included attention, working memory (digit span), writing– and visual–motor coordination, processing accuracy and speed, language acquisition and expression, and emotional health. In addition, among CCS, those with non-CNS tumors, with at least one parent of a higher educational level or a higher family income, scored significantly higher in the intelligence subscales than those with CNS tumors or parents of a lower educational level or lower income, respectively. In line with our findings, several studies have demonstrated lower intelligence scores for CCS (mainly ALL patients) than controls [4,18,19,20,21,22,23,24].

In a multicenter study, female pediatric ALL survivors exhibited significantly lower IQ in general than males [25]. In our study, the burden on female IQ as a whole was not observed, as females scored significantly lower than males only in a subscale of VIQ. However, significant differences in FSIQ and VIQ were detected between female CCS and controls, whereas male CCS retained their total IQ unaffected. Among children with ALL, females have demonstrated greater impairment of processing speed than males [26], which can be explained by developmental gender differences in cerebral myelination [27].

FSIQ, VIQ and PIQ scores were significantly lower in CNS tumor survivors than in non-CNS tumor survivors. Survivors of BT also had significantly lower FSIQ and PIQ than survivors of ALL or other types of cancer. This is in line with a meta-analysis of 22 studies highlighting serious intellectual impairment associated with BT [28]. Significantly lower speeds of oral/reading comprehension and non-verbal reasoning were observed in radiotherapy vs. non-radiotherapy-treated CCS, which can be explained by the negative impact of cranial–spinal radiation on information processing speed via insult to the right optic radiations [29].

Lower FSIQ has been associated with higher cranial radiation dose and time since radiation therapy [30], probably due to cerebro-cerebellar circuit lesions that modulate critical executive functions [31]. Our findings show that the radiation load and area (CNS vs. non-CNS) were associated with impaired PIQ, whereas birth weight and time free of cancer were positively correlated with PIQ and VIQ, respectively.

We also found worse scores of psychological/behavioral function for CCS than controls. Similarly, the Childhood Cancer Survivor Study [32] reported significantly more symptoms of depression and somatic distress in survivors than sibling controls, whereas in another study, 14 parents reported more attention deficits and behavioral difficulties in ALL survivors than reference norms.

In agreement with other studies [33,34,35], higher parental educational level and economic status were associated with better intellectual functioning of CCS.

Several limitations should be considered when interpreting the findings of this study. First, the study employed LAMDA, a Greek language-based assessment tool, which may limit direct comparability with studies using other neuropsychological instruments. In addition, functional neuroimaging techniques were not included, which could have provided complementary information regarding neurobiological correlates of cognitive outcomes.

The study sample was restricted to children and adolescents recruited in Greece, which may limit the generalizability of the findings to other populations. Furthermore, objective intellectual assessment prior to cancer diagnosis was not available, making it difficult to determine pre-morbid cognitive functioning.

Recruitment was based on the number of eligible CCS during the study period at the participating center. Although a prospective sample size calculation was not performed due to the limited pool of eligible survivors available at the study center, post hoc power analysis indicated adequate statistical power for detecting group differences, i.e., statistical power of 83%, which exceeds the commonly accepted threshold of 80% reported in the literature [36]. The 1:2 ratio of CCS (cases) to controls was selected to optimize the statistical power of the study, as recommended in the methodological literature on case–control designs [10].

The relatively modest sample size and the smaller numbers within some subgroup analyses (e.g., by tumor type or treatment characteristics) may limit the stability of these estimates, and such findings should therefore be interpreted with caution. In addition, the study involved multiple statistical comparisons across cognitive and psychosocial outcomes, increasing the possibility of type I error. Finally, although cases and controls were matched on key demographic characteristics, the possibility of residual confounding from unmeasured factors cannot be completely excluded. The selection of hospital-based controls rather than community-based controls may have influenced comparability and external validity.

## 5. Conclusions

Oncology advocacy efforts focused on children and adolescents have been successful in attending to their complex medical needs, while their unique cognitive and social–emotional requirements are often overlooked. The results of this study suggest that CCS in Greece experience significant developmental—cognitive and psychosocial—burden with potential impact on young CCS’ schooling, academic performance, and future work and employment.

Cognitive/educational support to child and adolescent CCS is imperative, as young brain plasticity offers a window of opportunity for educational interventions to help address CCS’ potential cognitive and educational difficulties. These findings may contribute to informing healthcare planning and prioritizing allocation of resources, such as direct educational assessments and interventions by special educators that are often not available in cancer centers.

Efforts should also aim to identify prognostic markers and refine toxic cancer treatments to investigate negative prognostic factors. Optimizing the quality of life of cancer survivors is a key area of the “Europe’s Beating Cancer Plan”, which aims to “limit the disruptive impact of cancer on the education of children and young people affected by cancer” and, via the new “Cancer Survivor Smart-Card”, provide long-term monitoring of outcomes, treatment toxicities, and psychological and educational support to cancer survivors.

## Figures and Tables

**Table 1:** Cognitive and learning scores of study participants.

		Total Sample (*n* = 219)	CCS (*n* = 70)	Controls (*n* = 149)	*p* [Effect Sizes]
Cognitive assessment (WISC-III)	FSIQ	101.18 ± 21.41	94.83 ± 22.43	104.38 ± 20.22	**0.004** [−0.456]
	VIQ	50.86 ± 13.03	47.08 ± 13.81	52.76 ± 12.25	**0.005** [0.444]
	IN	9.51 ± 3.27	8.63 ± 3.55	9.94 ± 3.04	**0.009** [−0.407]
	SM	11.16 ± 3.40	10.44 ± 3.41	11.53 ± 3.35	**0.039** [−0.322]
	AR	9.93 ± 3.39	9.22 ± 3.54	10.28 ± 3.27	**0.043** [−0.314]
	VC	9.95 ± 3.12	9.32 ± 3.11	10.26 ± 3.09	**0.049** [−0.306]
	CM	10.31 ± 2.97	9.49 ± 3.03	10.72 ± 2.86	**0.007** [−0.421]
	DS	9.62 ± 2.95	8.77 ± 3.11	10.05 ± 2.79	**0.006** [−0.441]
	PIQ	50.31 ± 11.06	47.70 ± 11.82	51.62 ± 10.45	**0.021** [−0.359]
	PC	10.07 ± 2.88	9.51 ± 2.90	10.35 ± 2.84	0.058 [−0.295]
	CD *	10.00 (4.00)	9.00 (4.00)	10.00 (5.00)	**0.033** [−0.327]
	PA	9.80 ± 2.70	9.32 ± 2.76	10.04 ± 2.65	0.083 [−0.269]
	BD	10.89 ± 3.36	10.49 ± 3.81	11.09 ± 3.11	0.252 [−0.178]
	OA	9.72 ± 2.91	9.41 ± 3.12	9.88 ± 2.79	0.299 [−0.161]
	SS	10.42 ± 3.66	9.10 ± 3.64	11.09 ± 3.50	**<0.001** [−0.562]
Learning assessment (LAMDA)	Word/picture recognition (accuracy) *	3.00 (0.50)	3.00 (0.50)	3.00 (0.50)	0.625 [0.071]
	Word/picture recognition (speed) *	3.50 (1.50)	3.00 (1.50)	3.50 (1.00)	0.062 [−0.350]
	Spelling (accuracy)	2.86 ± 0.96	2.73 ± 1.04	2.92 ± 0.91	0.217 [−0.195]
	Spelling (speed)	3.05 ± 0.98	2.89 ± 1.00	3.12 ± 0.96	0.118 [−0.235]
	Oral/reading comprehension (accuracy) *	3.00 (1.50)	3.00 (1.00)	3.50 (1.50)	0.052 [−0.290]
	Oral/reading comprehension (speed) *	3.33 (1.67)	3.00 (1.67)	3.67 (1.33)	**0.018** [−0.335]
	Morphosyntactic awareness (accuracy) *	3.00 (1.00)	3.00 (1.50)	3.50 (1.00)	**0.013** [−0.396]
	Morphosyntactic awareness (speed) *	3.00 (2.00)	2.75 (2.50)	3.50 (1.50)	**0.028** [−0.378]
	Vocabulary (accuracy) *	3.00 (1.50)	3.00 (2.00)	3.50 (1.00)	0.124 [−0.290]
	Vocabulary (speed) *	3.50 (1.50)	3.50 (2.00)	3.50 (1.50)	0.793 [−0.021]
	Short-term memory (accuracy) *	3.00 (2.00)	3.00 (2.00)	4.00 (1.00)	0.333 [−0.117]
	Non-verbal reasoning (accuracy) *	4.00 (1.00)	3.25 (1.50)	4.00 (1.00)	**0.008** [−0.316]
	Non-verbal reasoning (speed)	2.80 ± 1.13	2.70 ± 1.06	2.85 ± 1.16	0.397 [−0.127]
	Rhythm sensitivity (accuracy)	3.39 ± 0.82	3.39 ± 0.79	3.39 ± 0.86	0.993 [−0.002]

CCS, childhood cancer survivors; WISC-III, Wechsler Intelligence Scale for Children—Third Edition; FSIQ, full-scale Intelligence Quotient; VIQ, Verbal Scale IQ; IN, Information; SM, Similarities; AR, Arithmetic; VC, Vocabulary; CM, Comprehension; DS, Digit Span; PIQ, Performance Scale IQ; PC, Picture Completion; CD, Coding; PA, Picture Arrangement; BD, Block Design; OA, Object Assembly; SS, Symbol Search. Values are expressed as mean ± standard deviation (SD) or * median (interquartile range). *p*-value calculated using a *t*-test after the assumption of homogeneity of variance or * using the Mann–Whitney U test. Statistically significant differences are noted in bold.

**Table 2:** Cognitive and learning parameters of CCS according to type/area of cancer.

		Type of Cancer					Area of Cancer		
		BT (*n* = 19)	ALL (*n* = 25)	Lymphoma (*n* = 17)	Other (*n* = 9)	*p* [Effect Sizes]	CNS Cancer (*n* = 19)	Non-CNS Cancer (*n* = 51)	*p* [Effect Sizes]
Cognitive assessment (WISC-III)	FSIQ	82.06 ± 23.45	100.09 ± 20.19	93.69 ± 21.67	108.56 ± 15.65	**0.011** ^a,c^ [0.169]	82.06 ± 23.45	99.93 ± 20.08	**0.003** [−0.849]
	VIQ	41.56 ± 12.37	48.52 ± 14.15	47.08 ± 15.23	54.44 ± 10.89	0.126 [0.092]	41.56 ± 12.37	49.29 ± 13.86	**0.044** [−0.575]
	IN	7.11 ± 3.18	9.57 ± 3.82	8.23 ± 3.77	9.89 ± 2.20	0.100 [0.100]	7.11 ± 3.18	9.24 ± 3.54	**0.030** [−0.620]
	SM	9.78 ± 2.51	10.70 ± 3.28	10.00 ± 4.02	11.78 ± 4.38	0.501 [0.039]	9.78 ± 2.51	10.71 ± 3.70	0.254 [−0.274]
	AR	8.06 ± 3.52	9.30 ± 3.42	9.46 ± 3.93	11.00 ± 3.00	0.235 [0.069]	8.06 ± 3.52	9.69 ± 3.48	0.099 [−0.467]
	VC	8.39 ± 3.22	9.78 ± 3.12	8.92 ± 3.20	10.56 ± 2.51	0.294 [0.061]	8.39 ± 3.22	9.69 ± 3.02	0.135 [−0.422]
	CM	8.22 ± 2.62	9.22 ± 2.98	10.54 ± 3.57	11.22 ± 1.99	**0.045** ^b^ [0.126]	8.22 ± 2.62	10.00 ± 3.06	**0.034** [−0.604]
	DS	7.50 ± 2.68	8.70 ± 3.27	9.64 ± 2.20	10.44 ± 3.71	0.086 [0.108]	7.50 ± 2.68	9.30 ± 3.14	**0.038** [−0.597]
	PIQ	40.50 ± 14.05	51.57 ± 9.15	46.38 ± 10.37	54.11 ± 7.98	**0.005** ^a,c^ [0.193]	40.50 ± 14.05	50.58 ± 9.54	**0.010** [−0.918]
	PC	8.56 ± 2.91	10.52 ± 1.90	7.69 ± 3.43	11.44 ± 2.19	**0.002** ^d,e^ [0.224]	9.00(4.25)	11.00 (4.00)	0.056 * [−0.467]
	CD	6.56 ± 3.76	9.57 ± 3.54	10.85 ± 2.91	11.00 ± 2.00	**0.001** ^a,b,c^ [0.229]	6.56 ± 3.76	10.22 ± 3.13	**<0.001** [−1.106]
	PA	7.94 ± 2.73	10.00 ± 2.52	8.92 ± 2.10	10.89 ± 3.26	**0.025** ^c^ [0.145]	7.94 ± 2.73	9.87 ± 2.61	**0.011** [−0.727]
	BD	9.06 ± 4.53	11.17 ± 3.14	10.38 ± 4.25	11.78 ± 2.54	0.229 [0.070]	9.06 ± 4.53	11.07 ± 3.36	0.057 [−0.540]
	OA	9.00 (5.00)	10.00 (4.00)	11.00 (4.50)	11.00 (4.00)	0.403 * [0.045]	9.00 (5.00)	11.00 (4.00)	0.089 * [−0.439]
	SS	7.39± 3.57	9.39 ± 4.03	10.45 ± 2.30	10.11 ± 3.22	0.092 [0.106]	7.39 ± 3.57	9.81 ± 3.46	**0.016** [−0.695]
Learning assessment (LAMDA)	Word/picture recognition (accuracy)	2.69 ± 0.42	2.73 ± 0.46	2.82 ± 0.30	2.94 ± 0.46	0.444 [0.042]	2.75 (0.50)	3.00 (0.50)	0.266 * [−0.260]
	Word/picture recognition (speed)	2.86 ± 1.00	2.93 ± 0.79	3.21 ± 1.02	3.56 ± 0.63	0.220 [0.068]	2.86 ± 1.00	3.15 ± 0.87	0.259 [−0.315]
	Spelling (accuracy)	2.72 ± 0.94	2.57 ± 1.09	2.88 ± 1.01	2.89 ± 1.27	0.785 [0.017]	2.72 ± 0.94	2.74 ± 1.09	0.952 [−0.017]
	Spelling (speed)	2.50 (2.13)	3.00 (2.00)	3.00 (2.00)	3.50 (1.00)	0.277 * [0.061]	2.58 ± 1.07	3.01 ± 0.96	0.124 [0.431]
	Oral/reading comprehension (accuracy)	2.75 ± 0.90	3.09 ± 0.84	2.85 ± 0.93	3.00 ± 0.66	0.628 [0.027]	2.75 ± 0.90	2.99 ± 0.83	0.312 [−0.281]
	Oral/reading comprehension (speed)	2.61 ± 1.00	2.88 ± 1.02	2.90 ± 0.95	3.26 ± 0.86	0.445 [0.042]	2.61 ± 1.00	2.96 ± 0.96	0.200 [−0.358]
	Morphosyntactic awareness (accuracy)	2.53 ± 0.90	2.66 ± 0.89	3.12 ± 0.88	2.78 ± 1.03	0.263 [0.062]	2.53 ± 0.90	2.84 ± 0.92	0.215 [−0.346]
	Morphosyntactic awareness (speed)	2.44 ± 1.16	2.64 ± 1.07	2.62 ± 1.27	3.00 ± 1.20	0.715 [0.022]	2.44 ± 1.16	2.70 ± 1.15	0.430 [−0.220]
	Vocabulary (accuracy)	2.67 ± 0.99	3.30 ± 0.68	2.94 ± 1.01	2.89 ± 1.02	0.190 [0.073]	2.67 ± 0.99	3.09 ± 0.88	0.094 [−0.470]
	Vocabulary (speed) *	2.75 (2.00)	4.00 (1.50)	3.50 (2.25)	4.00 (1.50)	0.266 * [0.063]	2.75 (2.00)	4.00 (1.50)	0.114 * [−0.453]
	Short-term memory (accuracy)	2.94 ± 1.11	3.00 ± 0.98	3.00 ± 1.00	3.22 ± 0.97	0.926 [0.007]	2.94 ± 1.11	3.04 ± 0.97	0.728 [−0.097]
	Non-verbal reasoning (accuracy)	3.14 ± 0.94	3.05 ± 0.96	3.44 ± 0.75	2.83 ± 0.90	0.367 [0.049]	3.14 ± 0.94	3.15 ± 0.89	0.978 [−0.008]
	Non-verbal reasoning (speed)	2.86 ± 0.90	2.77 ± 1.05	2.15 ± 1.17	3.28 ± 0.83	**0.047** ^e^ [0.120]	2.86 ± 0.90	2.65 ± 1.12	0.468 [0.202]
	Rhythm sensitivity (accuracy)	3.00 (0.75)	4.00 (0.50)	2.50 (1.75)	4.00 (1.00)	**0.037** *^,d,e^ [0.282]	3.00 (0.75)	4.00 (1.00)	**0.039** * [−0.996]

WISC-III, Wechsler Intelligence Scale for Children—Third Edition; FSIQ, full-scale Intelligence Quotient; VIQ, Verbal Scale IQ; IN, Information; SM, Similarities; AR, Arithmetic; VC, Vocabulary; CM, Comprehension; DS, Digit Span; PIQ, Performance Scale IQ; PC, Picture Completion; CD, Coding; PA, Picture Arrangement; BD, Block Design; OA, Object Assembly; SS, Symbol Search; BT, brain tumor; ALL, acute lymphoblastic leukemia; CNS, central nervous system. For type of cancer, values are expressed as mean ± standard deviation (SD) and *p*-value calculated using analysis of variance (ANOVA) or * median (interquartile range) and *p*-value calculated using the Kruskal–Wallis test. After post hoc tests, statistically significant differences were found between ^a^ brain tumor—leukemia, ^b^ brain tumor—lymphoma, ^c^ brain tumor—other, ^d^ leukemia—lymphoma, ^e^ lymphoma—other. For area of cancer, values are expressed as mean ± standard deviation (SD) and *p*-value calculated using a *t*-test after the assumption of homogeneity of variance or * median (interquartile range) and *p*-value calculated using the Mann–Whitney U test. Statistically significant differences are noted in bold.

**Table 3:** YSR and CBC scales of CCS and controls.

		Children (YSR)			Parents (CBC)		
		CCS (*n* = 62)	Controls (*n* = 131)	*p* [Effect Sizes]	CCS (*n* = 62)	Controls (*n* = 131)	*p* [Effect Sizes]
Scales	Anxious/Depressed	58.42 ± 12.95	30.76 ± 35.00	**<0.001** [0.905]	72.77 ± 17.26	41.44 ± 40.01	**<0.001** [0.910]
	Withdrawn/Depressed	61.71 ± 14.62	27.96 ± 31.48	**<0.001** [1.210]	72.90 ± 16.33	39.84 ± 38.49	**<0.001** [1.000]
	Somatic Complaints	59.42 ± 14.13	30.11 ± 34.64	**<0.001** [0.963]	69.23 ± 18.68	37.13 ± 35.93	**<0.001** [1.020]
	Social Problems	56.76 ± 11.02	29.42 ± 33.38	**<0.001** [0.942]	68.10 ± 16.63	41.16 ± 39.47	**<0.001** [0.795]
	Thought Problems	61.61 ± 15.63	30.93 ± 35.49	**<0.001** [0.979]	67.82 ± 17.39	38.18 ± 36.72	**<0.001** [0.931]
	Attention Problems	57.29 ± 12.94	27.68 ± 31.30	**<0.001** [1.076]	63.42 ± 14.24	37.31 ± 35.54	**<0.001** [0.859]
	Rule-Breaking Behavior	54.82 ± 8.55	25.98 ± 28.90	**<0.001** [1.152]	64.23 ± 14.19	36.22 ± 34.52	**<0.001** [0.946]
	Aggressive Behavior	57.32 ± 13.10	28.10 ± 31.89	**<0.001** [1.043]	63.15 ± 16.21	36.93 ± 35.24	**<0.001** [0.860]
	Internalizing	43.76 ± 29.36	24.13 ± 33.58	**0.002** [0.604]	67.56 ± 28.67	37.70 ± 39.59	**<0.001** [0.819]
	Externalizing	33.61 ± 26.48	19.09 ± 26.85	**0.005** [0.543]	51.79 ± 26.59	32.03 ± 34.78	**<0.001** [0.610]
	Total Problems	36.00 ± 29.66	21.89 ± 31.27	**0.018** [0.458]	58.39 ± 27.85	34.80 ± 38.03	**<0.001** [0.672]
DSM-oriented scales	Depressive Problems	58.87 ± 11.12	29.60 ± 33.75	**<0.001** [0.998]	70.48 ± 17.97	40.15 ± 38.58	**<0.001** [0.908]
	Anxiety Problems	60.32 ± 14.96	30.65 ± 34.50	**<0.001** [0.975]	75.13 ± 17.63	41.86 ± 39.97	**<0.001** [0.966]
	Somatic Problems	59.97 ± 14.18	30.80 ± 35.35	**<0.001** [0.941]	67.03 ± 19.24	36.36 ± 35.06	**<0.001** [0.994]
	Attention Deficit/ Hyperactivity Problems	57.95 ± 12.40	28.45 ± 32.38	**<0.001** [1.041]	63.24 ± 14.50	36.34 ± 34.75	**<0.001** [0.902]
	Oppositional/Defiant Problems	57.61 ± 14.22	27.42 ± 30.81	**<0.001** [1.106]	61.87 ± 14.26	36.66 ± 34.82	**<0.001** [0.845]
	Conduct Problems	54.89 ± 9.96	26.64 ± 29.81	**<0.001** [1.090]	61.37 ± 15.01	35.07 ± 33.57	**<0.001** [0.908]
Disorders	Obsessive–Compulsive Problems	62.95 ± 17.10	31.52 ± 35.87	**<0.001** [0.987]	62.74 ± 15.85	36.18 ± 35.02	**<0.001** [0.878]
	Posttraumatic Stress Problems	60.68 ± 13.96	29.30 ± 33.71	**<0.001** [1.059]	72.94 ± 17.40	39.72 ± 38.64	**<0.001** [0.996]
	Positive Qualities	42.53 ± 26.16	25.05 ± 31.82	**0.001** [0.575]	69.61 ± 18.04	40.20 ± 38.97	**<0.001** [0.872]

CCS, childhood cancer survivors. Values are expressed as mean ± standard deviation (SD). *p*-value calculated using a *t*-test after the assumption of homogeneity of variance. Statistically significant differences are noted in bold.

**Table 4:** CCS’ cognitive and learning parameters according to parental education and family financial status.

		Parental Education			Family Financial Status		
		Parental Secondary or Lower (*n* = 40)	Education Higher (*n* = 26)	*p* [Effect Sizes]	≤20,000 € (*n* = 37)	>20,000 € (*n* = 20)	*p* [Effect Sizes]
Cognitive assessment (WISC-III)	FSIQ	89.84 ± 22.39	106.18 ± 17.09	**0.005** [−0.794]	92.57 ± 23.40	98.72 ± 17.31	0.330 [−0.285]
	VIQ	44.14 ± 13.32	54.50 ± 10.37	**0.003** [−0.842]	45.69 ± 13.28	49.67 ± 11.67	0.287 [−0.312]
	IN	7.57 ± 3.25	11.00 ± 2.85	**<0.001** [−1.106]	8.00(5.00)	10.00 (4.00)	**0.029** * [−0.761]
	SM	9.70 ± 2.84	12.23 ± 3.48	**0.004** [−0.818]	10.43 ± 3.09	10.33 ± 3.87	0.923 [0.028]
	AR	9.05 ± 3.81	10.14 ± 2.75	0.249 [−0.313]	9.49 ± 3.62	8.94 ± 2.71	0.580 [0.162]
	VC	8.73 ± 3.15	10.68 ± 2.25	**0.014** [−0.684]	8.71 ± 2.95	9.83 ± 2.31	0.167 [−0.407]
	CM	9.16 ± 3.02	10.41 ± 2.82	0.122 [−0.423]	9.23 ± 2.80	10.28 ± 2.99	0.212 [−0.367]
	DS	7.89 ± 3.10	10.29 ± 2.88	**0.006** [−0.793]	8.09 ± 3.30	10.24 ± 2.75	**0.025** [−0.686]
	PIQ	45.62 ± 12.79	51.68 ± 9.52	0.059 [−0.518]	46.80 ± 13.63	49.06 ± 8.69	0.527 [−0.185]
	PC	10.00(4.50)	11.00 (4.00)	0.624 * [−0.118]	9.54 ± 3.17	9.06 ± 2.82	0.585 [0.160]
	CD	9.03 ± 4.21	9.77 ± 2.91	0.467 [−0.197]	9.00 ± 4.52	9.72 ± 2.47	0.532 [−0.183]
	PA	8.78 ± 2.90	10.36 ± 2.44	**0.036** [−0.577]	9.17 ± 3.03	9.61 ± 2.62	0.604 [−0.152]
	BD	9.65 ± 3.82	12.05 ± 3.06	**0.015** [−0.674]	9.94 ± 3.90	11.44 ± 2.99	0.159 [−0.414]
	OA	9.00 (4.00)	11.00 (4.25)	0.066 * [−0.432]	11.00 (5.00)	9.00 (5.00)	0.662 * [0.090]
	SS	8.28 ± 3.95	10.62 ± 2.77	**0.011** [−0.657]	8.15 ± 4.11	10.47 ± 2.60	**0.018** [−0.630]
Learning assessment (LAMDA)	Word/picture recognition (accuracy)	2.68 ± 0.45	2.92 ± 0.32	**0.014** [−0.599]	2.72 ± 0.42	2.84 ± 0.41	0.316 [−0.287]
	Word/picture recognition (speed)	3.06 ± 0.86	3.13 ± 1.01	0.794 [−0.068]	3.11 ± 0.88	2.97 ± 1.10	0.616 [0.143]
	Spelling (accuracy)	2.65 ± 1.10	2.98 ± 0.89	0.196 [−0.321]	2.69 ± 1.10	3.00 ± 0.99	0.314 [−0.288]
	Spelling (speed)	2.89 ± 1.05	2.81 ± 0.93	0.774 [0.075]	2.88 ± 1.06	2.92 ± 0.90	0.873 [−0.045]
	Oral/reading comprehension (accuracy)	3.00(1.50)	3.50(0.88)	**0.032** * [−0.543]	2.79 ± 0.87	3.11 ± 0.59	0.167 [−0.398]
	Oral/reading comprehension (speed)	2.88 ± 1.03	2.79 ± 0.93	0.722 [0.092]	2.86 ± 0.99	2.82 ± 1.01	0.897 [0.037]
	Morphosyntactic awareness(accuracy)	2.59 ± 0.91	3.13 ± 0.82	**0.021** [−0.610]	2.64 ± 0.93	3.13 ± 0.91	0.065 [−0.533]
	Morphosyntactic awareness (speed)	2.60 ± 1.13	2.65 ± 1.21	0.879 [−0.039]	2.57 ± 1.12	2.71 ± 1.27	0.674 [−0.120]
	Vocabulary (accuracy)	2.88 ± 0.95	3.19 ± 0.78	0.177 [−0.348]	2.93 ± 0.97	3.08 ± 0.87	0.580 [−0.158]
	Vocabulary (speed) *	3.03 ± 1.08	3.17 ± 1.04	0.608 [−0.133]	3.08 ± 1.00	3.00 ± 1.09	0.778 [0.081]
	Short-term memory (accuracy)	3.00 (2.00)	4.00(1.00)	0.056 * [−0.502]	2.83 ± 1.06	3.21 ± 0.85	0.186 [−0.380]
	Non-verbal reasoning (accuracy)	3.03 ± 0.95	3.27 ± 0.81	0.293 [−0.274]	3.07 ± 1.01	3.16 ± 0.85	0.746 [−0.092]
	Non-verbal reasoning (speed)	2.71 ± 1.06	2.69 ± 1.14	0.929 [0.023]	2.85 ± 0.95	2.53 ± 1.26	0.340 [0.300]
	Rhythm sensitivity (accuracy)	3.37 ± 0.83	3.50 ± 0.76	0.703 [−0.162]	4.00 (1.00)	4.00(1.00)	0.473 * [−0.278]

WISC-III, Wechsler Intelligence Scale for Children—Third Edition; FSIQ, full-scale Intelligence Quotient; VIQ, Verbal Scale IQ; IN, Information; SM, Similarities; AR, Arithmetic; VC, Vocabulary; CM, Comprehension; DS, Digit Span; PIQ, Performance Scale IQ; PC, Picture Completion; CD, Coding; PA, Picture Arrangement; BD, Block Design; OA, Object Assembly; SS, Symbol Search; BT, brain tumor; ALL, acute lymphoblastic leukemia; CNS, central nervous system. Values are expressed as mean ± standard deviation (SD) and *p*-value calculated using a *t*-test after the assumption of homogeneity of variance or * median (interquartile range) and *p*-value calculated using the Mann–Whitney U test. Statistically significant differences are noted in bold.

**Table 5:** KIDSCREEN scales for CCS and controls.

		CCS (*n* = 66)	Controls (*n* = 144)	*p* [Effect Sizes]
Scales	Physical Well-being	18.74 ± 3.72	19.20 ± 3.41	0.380 [−0.131]
	Psychological Well-being	23.17 ± 4.45	22.95 ± 4.71	0.755 [0.046]
	Moods and Emotions *	10.00 (6.00)	12.00 (6.00)	**0.006** [−0.344]
	Self-Perception *	22.00 (6.00)	21.00 (5.00)	0.108 [0.257]
	Autonomy	17.64 ± 4.61	16.04 ± 4.90	**0.027** [0.331]
	Parent Relations and Home Life *	24.00 (8.25)	24.00 (7.00)	0.449 [0.141]
	Financial Resources	10.61 ± 3.13	10.88 ± 3.19	0.561 [−0.087]
	Social Acceptance (Bullying)	22.89 ± 5.11	23.43 ± 4.72	0.462 [−0.110]
	School Environment	22.71 ± 5.04	21.76 ± 5.34	0.227 [0.181]
	Social Support and Peers *	3.00 (2.00)	3.50 (1.00)	0.783 [−0.030]

CCS, childhood cancer survivors. Values are expressed as mean ± standard deviation (SD) or * median (interquartile range). *p*-value calculated using a *t*-test after the assumption of homogeneity of variance or * using the Mann–Whitney U test. Statistically significant differences are noted in bold.

## Data Availability

The original contributions presented in this study are included in the article. Further inquiries can be directed to the corresponding author.

## Abbreviations

The following abbreviations are used in this manuscript:

CCS	Childhood Cancer Survivors
CNS	Central Nervous System
BMI	Body Mass Index
BSA	Body Surface Area
CBCL	Child Behavior Checklist
YSR	Youth Self-Report
DSM	Diagnostic and Statistical Manual of Mental Disorders
HRQoL	Health-Related Quality of Life
MRI	Magnetic Resonance Imaging
WISC-III	Wechsler Intelligence Scale for Children—Third Edition
FSIQ	Full-Scale IQ
VIQ	Verbal IQ
PIQ	Performance IQ
IN	Information
SM	Similarities
AR	Arithmetic
VC	Vocabulary
CM	Comprehension
DS	Digit Span
PC	Picture Completion
CD	Coding
PA	Picture Arrangement
BD	Block Design
OA	Object Assembly
SS	Symbol Search
M	Mean
SD	Standard Deviation
ANOVA	Analysis of Variance
ALL	Acute Lymphoblastic Leukemia
